# The role of TGF-β and its crosstalk with RAC1/RAC1b signaling in breast and pancreas carcinoma

**DOI:** 10.1186/s12964-017-0175-0

**Published:** 2017-05-12

**Authors:** Catharina Melzer, Ralf Hass, Juliane von der Ohe, Hendrik Lehnert, Hendrik Ungefroren

**Affiliations:** 1 Biochemistry and Tumor Biology Lab, Department of Obstetrics and Gynecology, Hannover Medical School, Hannover, Germany; 2 Center of Brain, Behavior and Metabolism (CBBM), University of Lübeck, Campus Lübeck, Ratzeburger Allee 160, 23538 Lübeck, Germany; 3 First Department of Medicine, University Hospital Schleswig-Holstein (UKSH), Campus Lübeck, Ratzeburger Allee 160, 23538 Lübeck, Germany; 4 Department of General and Thoracic Surgery, UKSH, Campus Kiel, Kiel, Germany

**Keywords:** Breast cancer, Pancreas cancer, Tumor cell signaling, Tumor microenvironment, TGF-β, Rac1, Metastasis

## Abstract

This article focusses on the role of TGF-β and its signaling crosstalk with the RHO family GTPases RAC1 and RAC1b in the progression of breast and pancreatic carcinoma. The aggressive nature of these tumor types is mainly due to metastatic dissemination. Metastasis is facilitated by desmoplasia, a peculiar tumor microenvironment and the ability of the tumor cells to undergo epithelial-mesenchymal transition (EMT) and to adopt a motile and invasive phenotype. These processes are controlled entirely or in part by TGF-β and the small RHO GTPase RAC1 with both proteins acting as tumor promoters in late-stage cancers. Data from our and other studies point to signaling crosstalk between TGF-β and RAC1 and the related isoform, RAC1b, in pancreatic and mammary carcinoma cells. Based on the exciting observation that RAC1b functions as an endogenous inhibitor of RAC1, we propose a model on how the relative abundance or activity of RAC1 and RAC1b in the tumor cells may determine their responses to TGF-β and, ultimately, the metastatic capacity of the tumor.

## Background

### The dual role of TGF-β in cancer biology

TGF-β signaling has a central role in the progression towards a malignant state of stroma-rich carcinomas such as breast carcinoma and pancreatic ductal adenocarcinoma (PDAC) [1]. The crucial role of TGF-β signaling in carcinoma progression is highlighted by the fact that TGF-β is overexpressed in the tumor tissue and that overexpression correlates with poor prognosis [2]. Moreover, the TGF-β pathway has been identified as one of only four signaling pathways that are genetically altered (with at least one mutation) in 100% of PDAC [3]. However, the role of TGF-β during tumorigenesis is complex and somewhat paradoxical since in normal tissue and early-stage cancers it acts as a tumor suppressor by inhibiting epithelial cell cycle progression and promoting apoptosis, and only in late-stage counterparts it functions as a promoter by enhancing genomic instability, immune evasion, neoangiogenesis, cell motility, cancer invasiveness, and metastasis. This phenomenon has been termed the “TGF-β paradox” [4, 5] and is closely linked to the initiation of epithelial-mesenchymal transition (EMT) programs during tumor progression. Under the influence of TGF-β, the expression of which is increased in human carcinomas, particularly in those of the breast and the pancreas, tumor cells acquire a variety of phenotypes that endow these cells with a selective advantage to growing carcinomas, including i) enhanced motility; ii) greater resistance to cytotoxic agents, chemotherapeutics, and radiation treatments; and iii) enhanced expansion of cancer-initiating and stem-like cells. Currently, the molecular, cellular and microenvironmental mechanisms that enable post-EMT cancer cells to exploit the oncogenic activities of TGF-β remain largely unknown. Several excellent reviews have dealt with the issue of how TGF-β promotes EMT programs in late-stage carcinoma cells with some focussing on models of breast cancer [6–8] and pancreatic cancer [9, 10].

The conversion of premalignant cells to their metastatic counterparts via EMT programs dependent on TGF-β is facilitated by quantitative and qualitative changes of the tumor microenvironment. Here, TGF-β promotes the dialogue (direct and indirect interactions) of cancer cells with non-neoplastic cells such as stromal and immune cells and eventually their conversion into tumor-associated macrophages (TAMs) and cancer-associated fibroblasts (CAFs) from monocytes and fibroblasts, respectively [11]. In addition, TGF-β may directly control the amount and composition of the ECM, converting it into a fibrotic, proinflammatory tissue (desmoplastic reaction). The activated stroma consists besides tumor and non-neoplastic cells of a diverse array of proteins such as growth factors, matrix proteins, proteases, protease inhibitors, and integrins. TGF-β also favors hypoxia and modulates the physical properties of the ECM such as tissue/matrix compliance (e.g. tension and stiffness). These alterations directly impact the invasive capacity of the tumor cells and promote metastatic spread [11–14]. In addition to orchestrating the mesenchymal transition to a migratory phenotype at the single cell level, TGF-β signaling is also involved in a switch from collective movement to single-celled migration and prevention of TGF-β signaling reverts this migration type switch [15].

### Partial EMT and its relevance for invasion, metastasis, drug resistance and recurrence

**Fig. 1:**
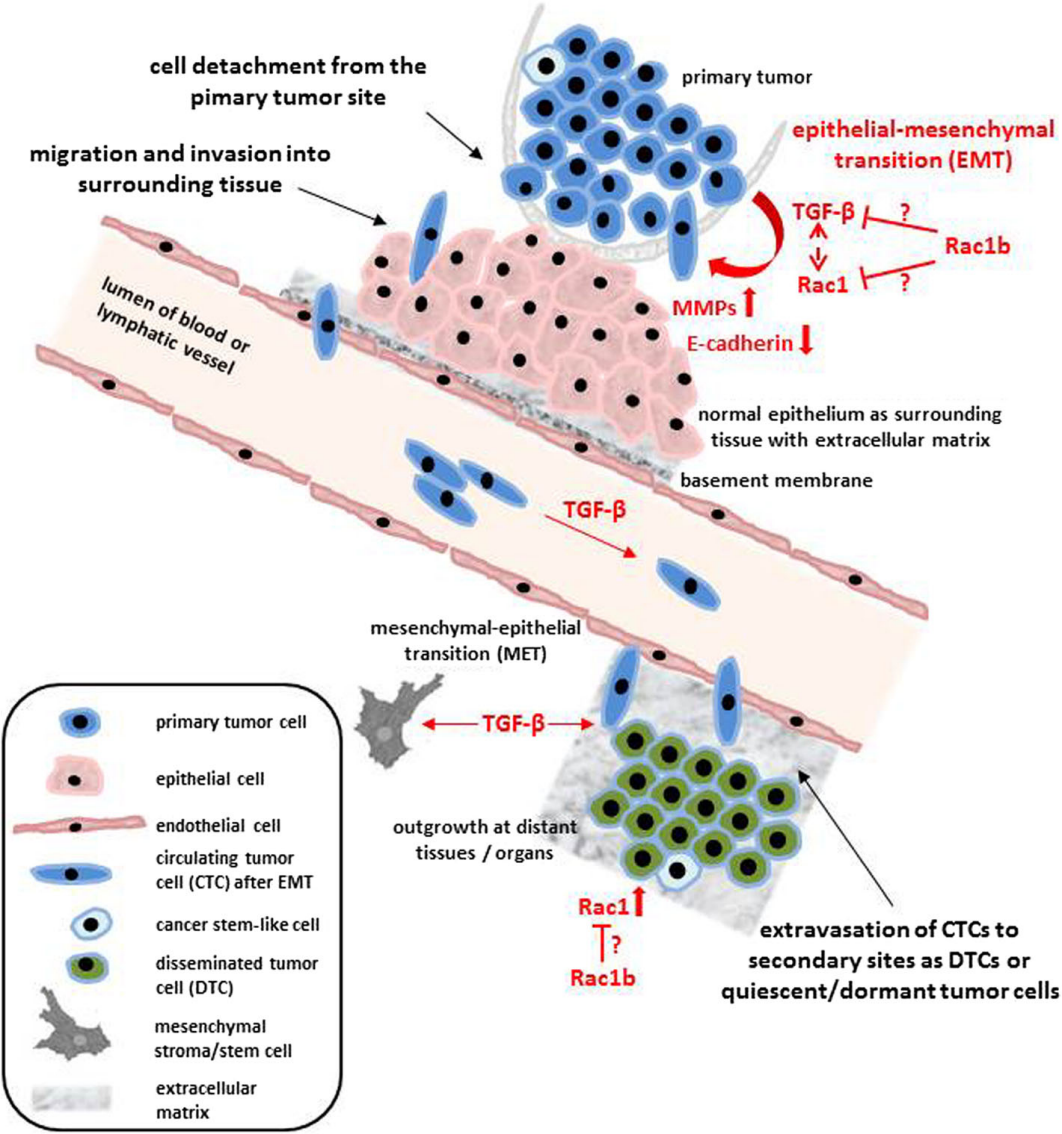
Schematic involvement of TGF-β and RAC1/RAC1b during metastatic steps. Metastasizing tumor cells from primary tumor site with self-renewing cancer stem-like cells undergo EMT and disseminate to distant organs (DTCs) after transendothelial migration as circulating tumor cells (CTCs) (modified according to Pantel and Brakenhoff [109]). TGF-β and RAC1/RAC1b play important roles during EMT and are also associated with further steps during metastasis. In particular, TGF-β promotes the dialogue of cancer cells with non-neoplastic cells of the tumor microenvironment such as epithelial, stromal and immune cells to promote EMT by down-modulation of E-cadherin expression and induction of matrix metalloproteinase (MMP) production and release. Moreover, TGF-β contributes to collective-to-single cell movement. In addition, increased TGF-β levels in the circulation were suggested to correlate with elevated appearance of CTCs and predominant formation of lung metastases in breast cancer patients leading to poor prognosis [110]. The RHO GTPase RAC1 is involved in the control of important cellular functions such as adhesion, motility, and proliferative capacity. Hyperactivation of RAC1 is detected in the majority of breast and pancreatic cancers in which RAC1 can transduce signals from different receptors, including those for TGF-β. RAC1b has been shown to negatively regulate RAC1 activity [85] as well as TGF-β1-dependent cell motility, SMAD2/3 C-terminal phosphorylation and TGF-β/SMAD-mediated transcription [93] which leads to the hypothesis that RAC1 and RAC1b have antagonistic roles in the regulation of TGF-β-induced EMT and MET

Cancers of the breast and the pancreas belong to the most aggressive tumor types due their high invasive and metastatic capacity [16]. Patients usually die from the consequences of tissue damage or dysfunction resulting from the spread and growth of metastases and relapse rather than complications caused by growth of the primary tumor. Metastasis is a highly complex process involving both morphological and functional alterations of metastasizing tumor cells. The metastatic cascade comprises several steps including cell detachment from the primary tumor site, migration and invasion into surrounding tissue, and extravasation to secondary sites as disseminated tumor cells after transendothelial migration and intravasation into blood and/or lymphatic vessels [17, 18] (Fig. 1).

The structural and functional alterations of metastasizing tumor cells from breast and pancreatic carcinomas result from genetic and epigenetic changes and are associated with conversion of epithelial cells into cells with a mesenchymal phenotype [17]. EMT is considered a prerequisite for tumor cells to become motile and invasive and eventually metastatic. The EMT process is induced by signals originating from the tumor microenvironment encompassing activation of diverse receptor tyrosine kinases (RTKs) via binding of epidermal growth factor (EGF), hepatocyte growth factor (HGF, c-Met) or fibroblast growth factor (FGF) [19] or receptor serine/threonine kinases via binding of TGF-β or bone morphogenetic proteins (BMPs). Among these, TGF-β is probably the most powerful inducer of EMT. At the cellular level, EMT is characterized by downregulation of E-cadherin, secretion of enzymes, e.g. matrix metalloproteinases (MMPs), and gain of mesenchymal marker expression, e.g. N-cadherin, vimentin, and fibronectin. These regulatory events result in alterations in cell-cell and cell-matrix adhesion, loss of cell polarity, degradation of the ECM and lead to enhanced cell-stroma interactions (see below), and increased migration and augmented invasiveness [20–22]. These alterations which in turn are facilitated by loss of E-cadherin and a reduction of tight junctions favor the initial phase of metastatic dissemination characterized by the detachment of individual carcinoma cells or small clusters with mesenchymal traits from the primary tumor mass [23]. This occurs particularly at the invasive front of the tumor and has been described as tumor budding [24]. Tumor buds are characterized by incomplete or partial EMT [24] and various EMT-inducing signaling pathways, such as those stimulated by TGF-β and Wnt are activated in tumor budding [25]. As a result of (partial) EMT induction, a group of so-called EMT transcription factors comprising Smads, SNAIL1, SNAIL2/SLUG, TWIST1/2, ZEB1/2, and AP-1 family members becomes activated [26] and for instance represses E-cadherin which is important for cell-cell adhesion [27]. The EMT process also induces the cancer cells to secrete large amounts of matrix proteins and pro-angiogenic and anti-inflammatory factors that ultimately result in desmoplasia/tissue fibrosis, neoangiogenesis and immune evasion, respectively.

Under normal conditions, detachment of epithelial and endothelial cells from the ECM leads to anoikis (apoptosis of anchorage-dependent cells) [28] and anoikis sensitivity is maintained by the epithelial specific cell polarity proteins and controlled in a cooperative manner by TGF-β, Wnt and Hippo pathways (reviewed in [29]). EMT induces resistance of tumor cells to anoikis and this resistance contributes to metastasis and is a defining property also of cancer stem cells. Transcription factors that meditate EMT can downregulate cell polarity-determining adhesion molecules. The resulting loss of correctly localized cell-polarity complexes alters signaling through TGF-β, Wnt, and Hippo signaling pathways and, in turn, reinforces the EMT phenotype [30, 31]. The failure of polarity proteins to localize correctly to the membrane during EMT relieves the sequestration of Smads, e.g. the inhibitory interaction between the crumbs polarity complex and Smad3 that is mediated by YAP and TAZ, and promotes TGF-β signaling, which, in turn, stabilizes the EMT phenotype and eventually enables tumor cells to evade anoikis [32, 33]. The cell polarity protein complex scribble enforces anoikis-driven luminal clearing in acini from normal breast epithelial (MCF10a) cells through RAC1 and JNK, implicating RAC1-JNK signaling in linking polarity with anoikis [34].

TGF-β-induced EMT has been associated tumor metastasis and disease recurrence [35], increased drug resistance [22, 36] and resistance to radiotherapy [37, 38]. However, the relationship between ionizing radiation and EMT is complex since ionizing radiation itself can induce - in a TGF-β-dependent fashion [39] – EMT and metastasis formation in cancer cells [40]. While EMT is generally considered a prerequisite for metastasis, two recent studies in genetically engineered mouse models of breast and pancreatic cancer have challenged this view. They found the contribution of EMT program to metastasis to be dispensable in contrast to the induction of chemoresistance [41, 42]. In addition to increasing resistance to chemical and physical insults, TGF-β may promote survival of the cancer cells by decreasing their sensitivity to intrinsic apoptosis inducers such as TRAIL [43], and by inducing fibrosis/desmoplasia which prevents the diffusion of chemotherapeutic drugs to the cancer cells within the tumor tissue [44]. Finally, EMT is thought to promote the generation and maintenance of an epithelial cancer stem cell (CSC) pool [26]. Indeed, our own studies have shown that treatment with rec. TGF-β1 increases EMT and stem cell marker expression in a PDAC-derived cell line [45]. In the host, TGF-β-driven EMT and CSC formation may increase the chance of formation of dormant cells in protected cancer stem cell niches that after initial remission eventually lead to later disease recurrence [46].

### TGF-β signaling and role in EMT

TGF-β signaling starts with binding of TGF-β to its receptors triggering an intracellular signal cascade that is either Smad-mediated or non Smad-mediated [47–49]. Smad-mediated TGF-β signaling pathways involve formation of a complex of receptor-activated SMAD2 or SMAD3 and SMAD4 (encoded by *DPC4*) that translocates into the nucleus and activates or represses transcription of TGF-β responsive genes [27]. Alterations of TGF-β signaling in breast and pancreatic tumors primarily affect the receptor-dependent Smad-mediated signaling pathway. The most characteristic alteration of PDAC is that of *DPC4*, which suffers from loss-of-function mutations or genomic deletion [50]. To exert the various tumor cell-autonomous and prometastatic functions, TGF-β through its receptors can also trigger non-Smad signal transduction via RHO-like GTPases RAC and RHO [51], MAPK and phosphoinositide 3-kinase (PI3K) pathways. Activation of both Smad and non-Smad pathways is required to induce EMT [52]. The complexity of TGF-β signaling in EMT induction is also reflected in the various interactions of TGF-β signaling with at least six other signaling pathways (RTK signaling, cytokine signaling, Wnt/β-catenin signaling, Notch signaling, Sonic hedgehog signaling, Hippo signaling) in regulating the expression and/or activity of transcription factors that elicit the EMT [26]. Crosstalk with RTK signaling involves common intracellular mediators with a known role in driving proliferation and cell motility/metastasis such as Ki-RAS [53], SRC [54], and p53 [55, 56], all of which have been implicated in TGF-β-induced EMT. TGF-β signaling has also been shown to promote metastatic colonization and mesenchymal-epithelial transition (MET) by inhibiting Twist1 [57] (Fig. 1).

### TGF-β-dependent and independent activation of RAC1 signaling

RHO GTPases have been widely implicated in tumorigenesis and metastasis and control a number of essential cellular functions including adhesion, motility, and proliferation. Unlike Ras proteins, which are frequently mutated in cancer, RHO and RAC proteins themselves are either overexpressed or deregulated rather than being mutated (with a notable exception [58]) leading to enhanced activities. Overexpression/hyperactivation of RAC1 is detected in the majority of breast and pancreatic cancers particularly in the tumor stroma [59] and is generally a consequence of enhanced upstream inputs from RTKs, PI3K or guanine nucleotide exchange factors (GEFs), or reduced RAC inactivation by GTPase-activating proteins (GAPs). Activated RAC1 can exert its function via multiple effectors such as p21-activated kinase 1 (PAK1), or via RAC1-dependent NADPH oxidases which generate reactive oxygen species (ROS). RAC1 and RAC1b can also affect mitochondrial ROS generation via cytochrome c. RAC1 is localized in the mitochondria of alveolar macrophages from pulmonary fibrosis patients and increases mitochondrial H_2_O_2_ generation in these cells. Mitochondrial import requires the C-terminal cysteine (Cys-189) of RAC1, which is post-translationally modified by geranylgeranylation. Furthermore, H_2_O_2_ generation mediated by mitochondrial RAC1 requires electron transfer from cytochrome c to another cysteine residue on RAC1 (Cys-178) [60]. Moreover, phosphorylation of RAC1b at Ser-71 by activated rho-associated protein kinase 1 (ROCK1) facilitates the interaction between Rac1b and cytochrome c leading to an increase in ROS levels, mitochondrial dysfunction, abnormal nuclear morphology and DNA double-strand breaks. The RAC1b-ROCK interaction may be crucial for progression of Hutchinson-Gilford progeria syndrome, a genetic disease with manifestation of an aging phenotype in childhood [61].

ROS are important signaling intermediates and are essential in the growth of pancreatic cancer [62]. In both breast and pancreatic cancer cells, RAC1 is a downstream effector of ERBB receptors and mediates migratory responses by ERBB1/EGF receptor (EGFR) ligands such as EGF or TGF-α and in breast cancer cells also by ERBB3 ligands such as heregulins. P-REX1 is activated by the PI3K product phosphatidylinositol 3,4,5-trisphosphate and Gβγ subunits, and integrates signals from ERBB receptors and G protein-coupled receptors. In breast cancer cells, the Rac-GEF P-REX1 seems to be an essential mediator of RAC1 responses [63]. Most notably, P-REX1 is highly overexpressed in human luminal breast tumors, particularly those expressing ERBB2 and estrogen receptor [63]. In addition, MST3 promotes proliferation and tumorigenicity through the VAV2/RAC1 signal axis in breast cancer [64] and RASAL2 activates RAC1 to promote triple-negative breast cancer progression [65]. Loss of the E3 ubiquitin ligase HACE1 results in enhanced RAC1 signaling contributing to breast cancer progression [66] while eIF2α-mediated downregulation of RAC1 signaling attenuates malignant phenotypes of breast cancer cells [67]. In PDAC progression the RAC1 GEF VAV1 has been shown to possess a role by acting synergistically with the EGFR to stimulate pancreatic tumor cell proliferation [68]. Mechanistically, the effects of VAV1 require its GEF activity and the activation of RAC1, PAK1, and NF-κB and involve CYCLIN D1 upregulation. For its proliferative effect VAV1 needs to be stabilized by dynamin 2, which also potentiates invasive migration of pancreatic tumor cells [69]. Likewise, the RAC1-specific GEF TIAM1 plays an important role in proliferation and invasion of pancreatic cancer cells [70]. Besides these GEFs other factors are able to target RAC1 in PDAC. BART inhibits pancreatic cancer cell invasion by RAC1 inactivation through direct binding to active RAC1 [71] and microRNA-124 (miR-124) suppresses RAC1 expression. Hypermethylation-mediated silencing of miR-124 RAC1 leads to RAC1 upregulation and consequently to PDAC progression and metastasis [72]. Interestingly, RAC1 was also found to be upregulated in CAFs in the primary tumor and in those residing in lymph node metastatic sites [73].

### The role of RAC1 and RAC1b in breast and pancreatic cancer

As mentioned above, RAC1 is activated by RTKs and its signaling mediators such as RAS-RAF-MEK-ERK but can also be activated by TGF-β receptors. Activation by RTK/RAS is particularly relevant in pancreatic cancer which is characterized by high mutation frequency (>90%) of *Ki-RAS* carrying an oncogenic mutation. Hence, RAC1 activity is high as a consequence of constitutively active Ki-RAS. Studies in mouse models of pancreatic cancer have highlighted the crucial role of Ki-RAS driven RAC1 signaling in the initiation and progression of this highly aggressive tumor entity; the pancreas-specific activation of *Ki-RAS* leads to acinar-to-ductal metaplasia (ADM) and formation of PanIN precursor lesions. In this model, the pancreas-specific ablation of *Rac1* abrogates the development of ADM, delays the formation of PanIN lesions, blocks progression to pancreatic cancer and increases survival [74]. However, deletion of the entire RAC1 gene is expected to also abrogate expression of RAC1b, an alternative splice product of *RAC1* which has been shown to possess different signaling properties (see below). Moreover, as outlined below in more detail, RAC1 and RAC1b have antagonistic functions in the regulation of TGF-β signaling. Given these caveats, the effects observed in this mouse model cannot be ascribed to RAC1 alone. Rather, results from mouse models are needed which allow for selective depletion of RAC1 or RAC1b. Attesting to a crucial role of *RAC1*, another study has shown that PI3K regulation of RAC1 is required for Ki-RAS-induced pancreatic tumorigenesis in mice [75]. In already established tumors, blocking RAC1 signaling in pancreatic cancer cells in vitro has led to reductions in cell proliferation, viability, and migration. In mice implanted with pancreatic tumors, intratumoral injections of an adenovirus expressing the RAC1-T17N mutant have also led to significant tumor growth inhibition.

Recent studies have also revealed an unexpected role for RAC1 in the response of cancer cells to DNA damaging agents. In breast [76] and pancreatic [77] cancer cells, RAC1 inhibition reduces survival [76] and blocks activation of a G2/M cell cycle checkpoint, respectively, and sensitizes pancreatic cancer cells to γ-irradiation [77]. G2/M cell cycle represents a mechanism that protects cells from the effects of irradiation and radiomimetic agents. In turn, carbon-ion irradiation suppresses migration and invasiveness of human pancreatic carcinoma cells MIAPaCa-2 via RAC1 and RHOA degradation [78]. Inhibition or inactivation of RAC1 decreases estrogen receptor levels [79], and in PTEN-deficient and insulin-like growth factor I receptor-overexpressing human breast cancer SKBR3 cells reduces Trastuzumab resistance [80].

RAC1b is a RAC1 isoform that is generated by alternative splicing from *RAC1* and that differs from RAC1 by the in-frame insertion of a short exon encompassing 57 nucleotides (exon 3b) immediately behind the switch II region of RAC1. This stretch of 19 amino acids is thought to confer constitutive activity upon RAC1b [81, 82], and not surprisingly, available studies so far suggest that RAC1b has different functional and signaling properties and to be unable to interact with RHO-GDI, to signal to PAK1 and JNK [83] and to activate the RelB pathway [84]. Unlike RAC1, activated RAC1b is unable to induce lamellipodia formation [83]. Interestingly, RAC1b has been shown to negatively regulate RAC1 activity. The expression of RAC1b in HeLa cells interferes with RAC1 activation by PDGF, leads to a reduction in membrane-bound RAC1 and promotes an increase in RHO activity. The antagonistic relationship between RAC1 and RAC1b perturbs the regulatory circuitry that controls actin cytoskeleton dynamics thereby leading to tumor-linked alterations in cell morphology and motility [85].

RAC1b overexpression has been described in breast, colon, and lung cancer. In lung adenocarcinoma RAC1b is upregulated in a significant fraction of tumor sections in correlation with mutational status of *Ki-RAS* [86]. Studies with RAC1b transgenic mice to evaluate the role of RAC1b during tumor progression in breast and pancreatic cancer are not available yet, although using a lung adenocarcinoma mouse model, in which the expression of RAC1b can be conditionally activated, expression of RAC1b alone was insufficient to drive tumor initiation [86]. However, the expression of RAC1b synergized with an oncogenic allele of *Ki-Ras* resulting in increased cellular proliferation and accelerated tumor growth.

Activation of RHO-GTPases and particularly RAC1 is a key step in the mechanism of EMT and a likely contributor to tubulointerstitial fibrosis [87] and MET during somitic segmentation [88]. Interestingly, the mechanical rigidity/matrix stiffness of the (tumor) microenvironment plays a crucial role in the promotion of EMT by controlling the subcellular localization and downstream signaling of RAC1 and RAC1b. Soft substrata with compliances comparable to that of normal mammary tissue are protective against EMT, whereas stiff substrata with compliances characteristic of breast tumors promote EMT. In cells cultured on stiff substrata or in collagen-rich regions of human breast tumors, RAC1b localizes to the plasma membrane where it forms a complex with NADPH oxidase and promotes the production of ROS, expression of SNAIL, and activation of EMT program. In contrast, soft substrata inhibit the membrane localization of RAC1b and subsequent redox changes [89]. In rigid microenvironments, RAC1b upregulation and translocation to the cell membrane, and induction of ROS and promotion of EMT is induced by MMP3. This EMT response in MMP3-treated cells is suppressed by the basement membrane protein laminin, while it is promoted by fibronectin. These ECM proteins regulate EMT via interactions with their specific integrin receptors. α6-integrin sequesters RAC1b from the membrane and is required for inhibition of EMT by laminin, while α5-integrin maintains RAC1b at the membrane and is required for the promotion of EMT by fibronectin [90].

Both, MMP3 and RAC1b are expressed in PDAC cells and their expression was found to be associated with all tumor stages, whereby the subcellular distribution of RAC1b in PDAC is accompanied by the patient outcome [91]. In line with its ability to mediate MMP3-induced EMT and genomic instability via ROS production in certain microenvironments (see above) RAC1b can increase malignant transformation of breast cancer cells [92] and probably also of PDAC cells [91]. Since RAC1b negatively regulates TGF-β1-induced cell migration in pancreatic cells [93], it is conceivable that it also controls TGF-β1-dependent EMT in a negative fashion (see below).

Activation of RHO-GTPases including RAC1 results in cytoskeletal changes, lamellipodia and filopodia formation that increase cell motility [94, 95] and has been implicated in different forms of invasive motility. Whereas single cell movement is implemented as mesenchymal (slower migratory phenotype) or amoeboid cell invasion (faster migratory phenotype), collective invasion is only performed in a mesenchymal cell migration manner [17]. There is evidence that RAC1 induces mesenchymal elongated movement. In melanoma cells, mesenchymal migration was driven by activation of RAC via a complex of a Rac guanine nucleotide exchange factor DOCK3 and the adaptor protein NEDD9 (melanoma metastasis gene). Additionally, RAC is inactivated during amoeboid movement [96, 97].

The blood and lymphatic systems represent two possible routes for metastatic spread [98]. Lymphatic capillaries are thin-walled and consist of single endothelial cell layers which are not covered by pericytes or smooth muscle cells and do not exhibit a basement membrane in comparison to blood vessels [99]. RAC1 and VEGF have been attributed a central role in transendothelial migration. Lung cancer cell-secreted VEGF activated endothelial RAC1 through VEGFRs/PI3Kβ signaling cascade and thereby increased human umbilical vein endothelial cell permeability and transendothelial movement [100].

### RAC1 and RAC1b as antagonistic modulators of TGF-β signaling

Like TGF-β, RHO GTPases and particularly RAC1 fulfil specific functions in the metastatic cascade that are either TGF-β-dependent (when RAC1 acts a signal transducer in non-canonical TGF-β signaling) or independent of this growth factor (see above and Fig. 1).

RAC1 mediates the oncogenic effects of growth factor receptors, particularly those of the ERBB group and is an effector protein of Ki-RAS. In the light of the crucial roles of both TGF-β and RAC1 in tumor growth and various stages of the metastatic process (Fig. 1), it was not unexpected that RAC1 can also transduce signals from other receptors, e.g. those for TGF-β which have serine/threonine kinase activity. We and others have shown that RAC1 can be activated by TGF-β/ALK5 and is itself involved in intracellular signal transduction by promoting the C-terminal phosphorylation/activation of SMAD2 [101] and p38 MAPK [102].

In the course of analyzing the role of RAC1 in TGF-β -induced cell migration and invasion, expression of RAC1b was noted in various PDAC cell lines by qPCR and immunoblot analysis as well as in ductal cells in PDAC tissue from patients using immunohistochemistry [93]. In the light of the high structural similarity of RAC1 and RAC1b, both proteins were expected to be functionally equivalent and to both promote TGF-β1-induced migration. Surprisingly, however, depleting cells of RAC1b by RNA interference strongly enhanced the sensitivity of the cells to the pro-migratory effect of TGF-β1. Conversely and in agreement with the RNA interference data, stable ectopic overexpression of RAC1b diminished the TGF-β effect on cell migration in two PDAC cell lines [93]. When studying intracellular mediators of TGF-β signaling in RAC1b-depleted cells, enhanced C-terminal phosphorylation of SMAD2 and SMAD3 along with enhanced transcriptional activity from TGF-β/Smad-responsive reporter genes was noted in response to TGF-β1 stimulation, suggesting that RAC1b is a negative regulator of Smad signaling [93]. Moreover, other prominent responses to TGF-β1 such as EMT-associated changes and growth inhibition may also be affected by RAC1b in a negative fashion. Since both TGF-β responses are promoted by RAC1, it appears conceivable that RAC1 and RAC1b control TGF-β responses in cancer cells in an antagonistic manner with RAC1b acting as an endogenous inhibitor of RAC1. An opposing relationship was also observed by Nimnual and colleagues who provided evidence that RAC1b negatively regulates (PDGF and EGF-induced) RAC1 activity that leads to a reduction in membrane-bound RAC1 and promotes an increase in RHO activity [85].

RAC1b has been observed to inhibit neurotrophin 3 stimulated MEK-ERK1/2 signaling in human bone marrow-derived stromal cells [103]. Since ERK1/2 activation is crucial for TGF-β-induced EMT in PDAC cells [104], RAC1b may inhibit TGF-β dependent EMT in part through suppression of MEK-ERK1/2 signaling.

**Fig. 2:**
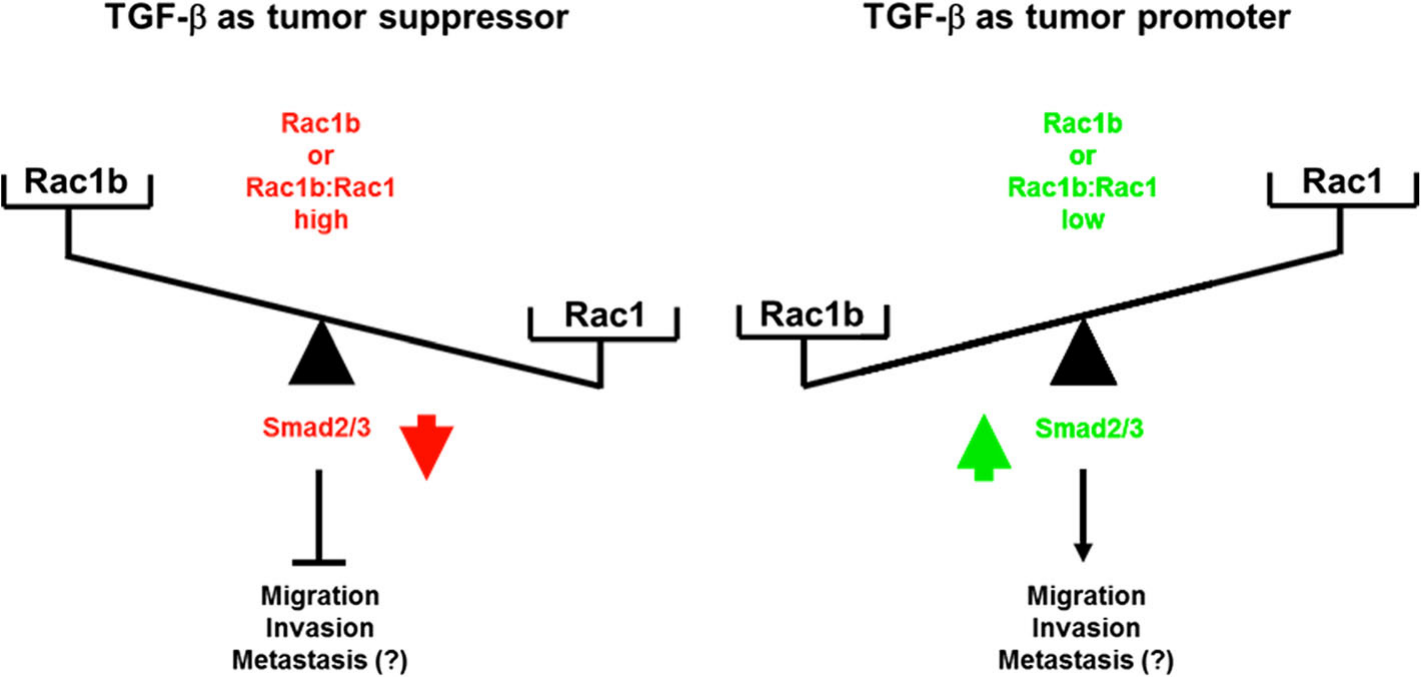
The dual role of TGF-β in tumor progression and its association with RAC1b and RAC1 expression. Depending on the stage of cancer progression, TGF-β can act either as tumor suppressor (left panel) or tumor promoter (right panel) by inhibiting or enhancing, respectively, cell migration, invasion, and metastasis, through the Smad signaling pathway. This phenomenon is known as the “TGF-β paradox”. Early-stage tumors with high RAC1b or a high RAC1b:RAC1 ratio are less invasive and metastatic due to functional inhibition of RAC1 (*left-hand side*), while advanced tumors expressing little RAC1b or maintaining a low RAC1b:RAC1 ratio eventually become more invasive and metastatic due to a preponderance of tumor-promoting RAC1 (right-hand side). Hence, the relative expression and activity of RAC1b and RAC1 may ultimately determine the tumor cells’ response to TGF-β during tumor progression. It should be noted that the tumor-suppressive effect of Rac1b is specific for TGF-β since in response to other EMT-inducers, such as MMP3, Rac1b can *increase* malignant transformation [92]. The red arrow indicates inhibition and the green arrow activation of SMAD2 and SMAD3 (SMAD2/3) activity

Based on their opposing effects, alterations in the ratio of RAC1b:RAC1 expression and/or activity may thus represent a potential tool for the tumor to modulate net TGF-β1 signaling activity. Tumors which express little RAC1b or maintain a low RAC1b:RAC1 ratio may become more aggressive and metastatic due to a preponderance of tumor-promoting RAC1 (Fig. 2). Indirect evidence for this was obtained from patient data demonstrating that high RAC1b expression in tumor cells in situ was associated with longer survival [95]. In contrast, the pro-invasive/pro-metastatic effects of RAC1 are neutralized in tumors with high RAC1b (Fig. 2). The (indirect) blocking of Smad activation and thus the growth-promoting function of RAC1b is negligible because the Smad pathway is already non-functional at these later stages in the majority of tumors due to loss-of-function mutations in *DPC4* or other alterations. The functional antagonism of RAC1b and RAC1 in controlling TGF-β signaling in conjunction with appropriate changes in their relative activities during tumor progression also represents a potential mechanism to explain the TGF-β paradox (Fig. 2).

### Therapeutic implications of differential inhibition of RAC1/RAC1b rather than the TGF-β receptors for the oncogenic potential of TGF-β in late-stage carcinomas

TGF-β signaling in cancer is considered a prominent target for a potential therapeutic approach in oncology [105]. Commonly used TGF-β pathway inhibitors acting at the ligand or receptor level such as TGF-β2 siRNA, neutralizing antibodies to the ligand or type II receptor, or small molecules such as SB431542 which block the kinase domain of ALK5 have now entered clinical trials [105].

In breast cancer, TGF-β blockade has been shown to restore the chemotherapeutic response through alleviation of desmoplasia [44]. However, more generalized inhibition strategies in current applications appear insufficient and thus not suitable for selectively targeting a specific TGF-β response(s). Moreover, blocking TGF-β signaling in both tumor and neighboring non-malignant cells may result in serious side effects. Rather, selective targeting of proteins that mediate specific pro-oncogenic responses, as a result of mutation or deregulation, would be desirable. Ki-RAS represents a good example for such a target. Unfortunately, efforts to target Ki-RAS in tumors pharmacologically using small molecule inhibitors have not been successful so far. Consequently, an alternative strategy focuses on downstream signaling effectors of the Ras pathway. Ki-RAS has four effectors that all play a role in cancer development: the MAPK pathway, the PI3K pathway, Ral guanine nucleotide dissociation stimulator (Ral-GDS), and RAC1 [106].

Above mentioned findings have revealed the potential value of RAC1 pathway inhibition as an attractive target for cancer therapy in part by sensitizing tumor cells to radio- and chemotherapy.

Given the crucial role of RAC1 in survival and RAS-mediated transformation, and of RAC1 and RAC1b in modulating the TGF-β pathway, therapeutic targeting of TGF-β signaling in cancer cells with inhibitors of RAC1 and/or RAC1b provides an exciting perspective. It may be a feasible therapeutic option to shift the signaling outcome from pro- to anti-oncogenic properties and to block malignant features while simultaneously maintaining or restoring beneficial functions of this growth factor.

Considering the promising results from in vitro and in vivo studies, novel inhibitors of RAC1 are currently evaluated preclinically as chemotherapeutic agents in metastatic breast cancer [107]. Moreover, novel drugs targeting the RAC1-GEF interaction are currently being developed using a rational design approach followed by evaluation for their anti-cancer properties in highly aggressive breast cancer cell lines [108]. The next step in the development of RAC1 pathway inhibitors will be the testing of second generation RAC1/PAK blockers. The first small molecules of this pathway were NSC23766 and EHT-1864. Second generation compounds that block RAC1 at the lower micromolar range include Ehop-016 and AZA1. Like PAK kinase inhibitors, these drugs have been reported to significantly reduce tumor growth in mouse models of breast, prostate, and brain cancer but have not yet been tested in preclinical models of pancreatic cancer. Unfortunately, all of the above mentioned agents have not been evaluated for their activity against RAC1b which, however, is important since RAC1b displays different signaling properties [83, 84]. Such test would contribute to clearly distinguish effects of RAC1b from those of RAC1.

## Conclusions

TGF-β plays a crucial role during EMT and metastasis, particularly in breast and pancreatic carcinoma. This is supported in part by RAC1 whereby TGF-β and RAC1 can exhibit distinct molecular interplays. Moreover, a potential functional antagonism may be displayed by RAC1b versus RAC1 in controlling TGF-β signaling in conjunction with appropriate changes in relative activities during tumor progression. With respect to potential pharmacological targets it will be interesting to follow effects of these agents in the treatment of breast and pancreatic cancers and whether they interfere with classical TGF-β responses. In the light of the intimate crosstalk of RAC1/RAC1b and TGF-β signaling in various tumor cell responses and the crucial role of TGF-β in driving the malignant process in both cancer types, it is conceivable that part of the novel RAC1 compound’s efficacy is due to differential inhibition of pro-oncogenic TGF-β responses.

## Abbreviations

ADM: Acinar-to-ductal metaplasia
CSC: Cancer stem cell(s)
EGF(R): Epidermal growth factor (receptor)
EMT: Epithelial-to-mesenchymal-transition
GAP: GTPase-activating protein
GEF: Guanine nucleotide exchange factor
MAPK: Mitogen-activated protein kinase
MET: Mesenchymal-to-epithelial-transition
MMP: Matrix metalloproteinase
PDAC: Pancreatic ductal adenocarcinoma
ROCK: Rho-associated protein kinase
RTK: Receptor tyrosine kinase
